# The GUD technique: glandar urethral disassembly. An alternative for distal hypospadias repair

**DOI:** 10.1590/S1677-5538.IBJU.2018.0835

**Published:** 2020-09-02

**Authors:** Antonio Macedo, Sérgio Leite Ottoni, Gilmar Garrone, Riberto Liguori, Ricardo Marcondes Mattos, Marcela Leal da Cruz

**Affiliations:** 1 Departamento de Urologia CACAU São PauloSP Brasil Departamento de Urologia, CACAU-NUPEP, São Paulo, SP, Brasil;; 2 Departamento de Pediatria Universidade Federal de São Paulo São PauloSP Brasil Departamento de Pediatria, Universidade Federal de São Paulo, São Paulo, SP, Brasil

**Keywords:** Hypospadias, Urethra, Circumcision

## Abstract

**Introduction:**

We present an alternative procedure for distal hypospadias consisting of urethral mobilization and partial glandar disassembly, namely GUD (glandar urethral disassembly) technique.

**Materials and Methods:**

A subcoronal circumcision exposes distal dysplastic urethra. We incise the Buck´s fascia on both sides of urethra releasing it partially from the corpora. We keep a thin bridge of urethral plate to the glans and disassembly almost completely the glans from the corpora, except for the bridge. The glans is incised creating two wide wings that are extremely mobile. The urethra is mobilized, advanced and sutured to the tip of the glans. The glans wings embrace the distal urethra producing a conical glans.

**Discussion:**

The concept of urethral mobilization has been reported and popularized by Koff in the literature to correct distal hypospadias. One of the limitations of this procedure is the risk of urethral retraction due to extensive proximal dissection. We got inspiration from Mitchell and Bagli’ s work of penile disassembly in epispadias to develop the GUD concept. We adopt minimal urethral mobilization mainly in glandar/proximal penile shaft and complete deconstruction of the glans, detaching the corpora from the glans and rotating the wide glans wings to embrace the urethra. Therefore we avoid suture urethroplasty and refurbish the glans to a better conical shape.

**Conclusion:**

We are convinced that this operation can be regarded as a genuine alternative to distal hypospadias (coronal and subcoronal) but should not be addressed to midshaft forms.

## INTRODUCTION

Distal hypospadias can be treated by different techniques although TIP repair has been regarded as the preferred choice by many authors ( 1 ). Systematic literature review reports urethroplasty complications in approximately 7% of distal TIP repairs, even though other authors report higher complications rate ( 2 ).

In this video we want to describe an alternative procedure for distal hypospadias consisting of urethral mobilization and partial glandar disassembly (the GUD technique).

## MATERIALS AND METHODS

**Figure 1:**
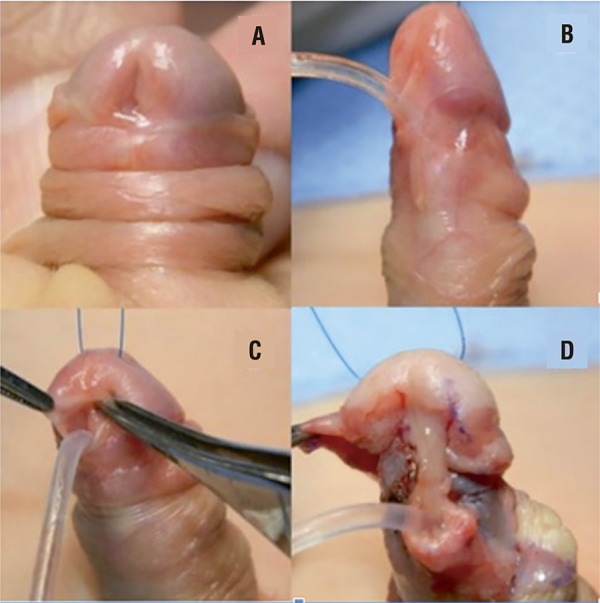
GUD Technique – Partial Glandar Disassembly.

A subcoronal circumcision is performed and the entire penis shaft is degloved exposing distal urethra. We incise the Buck´s fascia on both sides lateral to distal urethra releasing it from the corpora. From this standpoint, we dissect the glans and disassembly it almost completely from the tip of corpora, except for a bridge of tissue similar to urethral plate connecting the urethra to the tip of glans. The urethra is mobilized and the glans incised creating two very mobile wings that allow a thorough refurbishment of the glans ( Figure-1 ). No major bleeding from the spongious tissue occurs when the disassembly process respects the anatomy and the penis is compressed with a tourniquet on its base.

**Figure 2:**
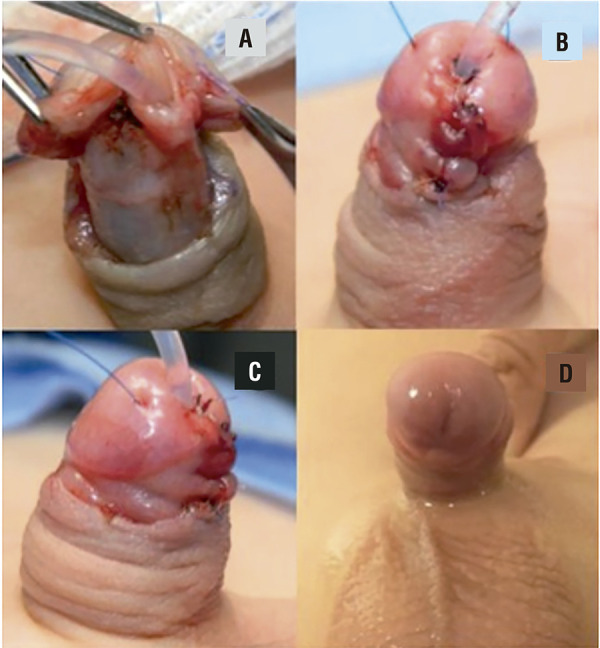
GUD technique: final aspect.

We suture the urethra to the glans with interrupted 7.0 PDS sutures. The spongious tissue of the distal urethra is sutured in the midline with 6.0 PDS. The lateral borders of the glans are brought together over the urethra with subcutaneous/subepithelial 5.0 PDS sutures concluding the glans reconstruction. A 6F silicone catheter is left indwelling for 7 days just to avoid voiding discomfort in case of glans edema ( Figure-2 ). A Tegaderm dressing is applied to the shaft of the penis concluding the procedure.

## DISCUSSION

Koff et al. published a modification of the Barcat technique known as extensive urethral mobilization and confirmed excellent cosmetic and functional results on 168 patients with only 3.5% of the patients requiring reoperation ( 3 ). This technique has been also reproduced by several other groups.

**Figure 3:**
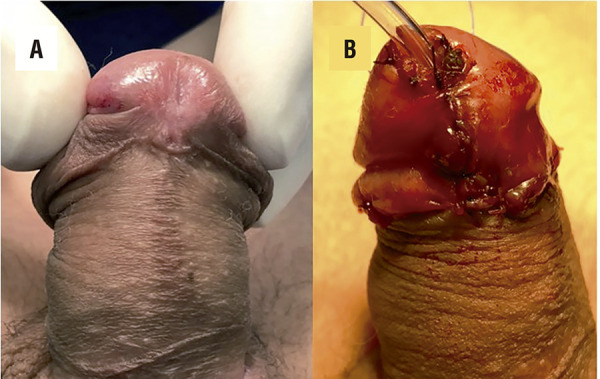
GUD technique – Detail of conic aspect of the glans.

Mitchell & Blagi ( 4 ) and Perovic et al. ( 5 ) reported on complete penile disassembly for epispadia repair as a way to complete release of the rotation of the penis and treat dorsal chordee bringing the urethra to a more functional location. We combined both procedures to propose the GUD technique. The rationale for this procedure is to avoid suture urethroplasty, except sutures to reposition the urethra distally and without tension. The aggressive partial glandar disassembly is the major difference compared to other modifications of Koff’s technique, that rely mostly in proximal mobilization of the urethra up to the bulbar area. Those procedures incise the glans to get two wings but do not disconnect it from corpora totally. We are convinced the GUD technique combines up-mobilization of the urethra with down mobilization and rotation of glans wings and the result is a conical and better cosmetic glans ( Figure-3 ).

## CONCLUSIONS

We are convinced that this operation can be regarded as a genuine alternative to distal hypospadias (coronal and subcoronal), but do not recommend it for midshaft forms.
